# Posterior vault distraction technique: how I do it

**DOI:** 10.1007/s00381-021-05203-x

**Published:** 2021-05-25

**Authors:** Niina Salokorpi, Leonid Satanin, Ivan Teterin, Juha-Jaakko Sinikumpu, Willy Serlo

**Affiliations:** 1 Department of Neurosurgery, Oulu University Hospital, P.O. Box 21, 90029 OYS Oulu, Finland; 2 Medical Research Center, Oulu University Hospital, Oulu, Finland; 3 Research Unit of Clinical Neuroscience, Oulu University Hospital and University of Oulu, Oulu, Finland; 4 Department of Paediatric Neurosurgery, Moscow Burdenko Neurosurgery Institute, Moscow, Russian Federation; 5 Department of Children and Adolescent, Oulu University Hospital, Oulu, Finland; 6 PEDEGO Research Group, Oulu University Hospital and University of Oulu, Oulu, Finland

**Keywords:** Posterior cranial vault distraction, Distraction devices, internal, Craniosynostosis, Surgical technique, Craniofacial malformations

## Abstract

Posterior cranial vault distraction is an effective technique when a significant increase in the intracranial volume is required in patients with craniosynostoses. This technique has been proven to be safe and time saving and usually is associated with low perioperative morbidity as well as low intraoperative bleeding. Herein a technique is presented starting from the preoperative planning, describing the surgical steps of the operation and the postoperative distraction protocol used by the authors. The authors present important tips and tricks aiming to minimise complications and undesired events.

## Introduction

The aim of posterior cranial vault distraction (PCVD) is to gain a significant increase in intracranial volume, with acceptable cosmetic results. This technique results in an increase in intracranial volume of about 25–30% in a controlled fashion and with minimal risk for serious perioperative complications [4, 12].

PCVD has been found to be suitable for patients from 3 months to over 10 years of age suffering from a large variety of conditions causing restriction of skull growth [4, 12]. There are no absolute contraindications for this procedure, except in cases with significant occipital venous anomalies [3] and it can be done repeatedly, if necessary [18].

## Presurgical assessment, surgical armamentarium, and anaesthesiologic considerations

### Preoperative studies

**Fig. 1:**
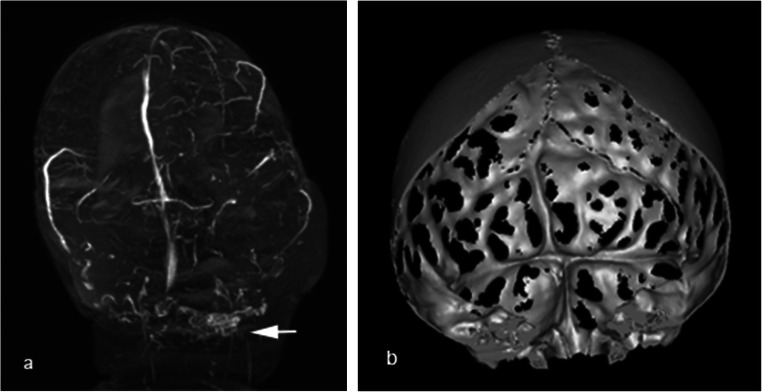
Anomalous anatomy in patients with craniosynostoses: **a** abnormal venous anatomy according to MR angiography images. **B** Appearance of the inner surface of the calvaria in a patient with Crouzon syndrome, 3D reconstruction of the preoperative CT scan

A three-dimensional (3D) skull computer tomography (CT) is required preoperatively. When evaluating the images, attention should be paid to the shape of the skull, patency of the sutures, presence of bony ridges, and location of the possible bony defects caused by increased intracranial pressure or due to previous operations (Fig. 1b). If vascular anomalies like sinus pericranii, stenosis of venous sinuses, collateral emissary veins, and persistence of embryologic sinuses are suspected, magnetic resonance angiography (MRA) should be performed (Fig. 1a). For example, in patients with stenosis of the venous sinuses, a significant amount of cerebral venous blood could be drained into the subcutaneous veins [17]. This could increase the risk of significant intraoperative bleeding [10] and life-threatening intracranial pressure (ICP) increase postoperatively due to altered cerebral venous drainage [3, 16] and potentially cause vascular complications during the activation period due to traction on anomalous venous structures.

### Preoperative planning

To achieve the desired result by the PCVD, preoperative planning should be done individually for each patient. In syndromic patients, all possible operations and treatments that the patient could need in the future should be taken into account. If such a patient will need shunting before PCVD, the shunt should be inserted into the posterior horn of the lateral ventricle to avoid conflict with the distractor devices later, when the PCVD will be performed (Fig. 7c). When planning the surgical approach, any future necessity for operations on anterior parts of the skull should be kept in mind. One should already at this stage keep in mind the necessity of removing the distraction devices afterwards.

**Fig. 2:**
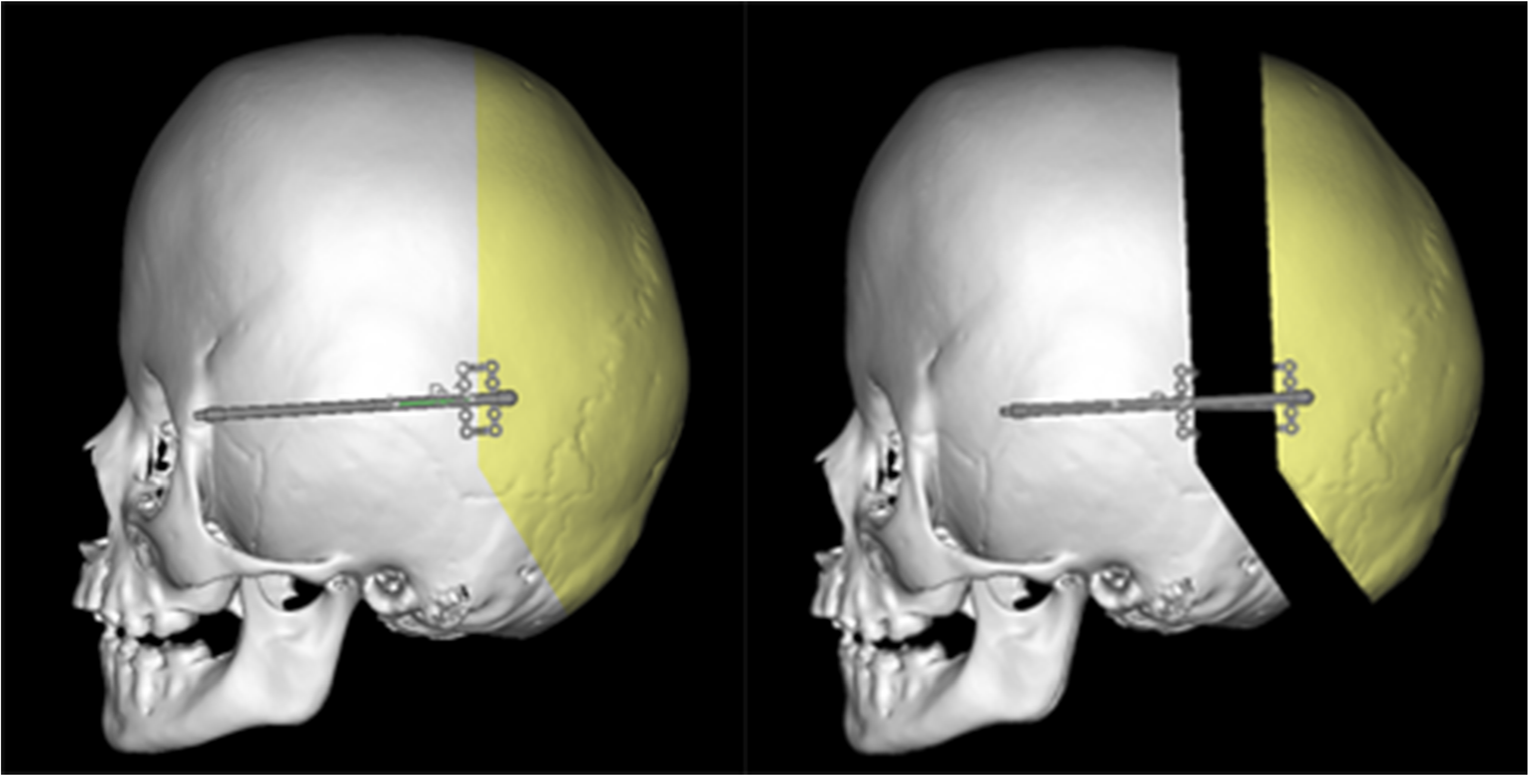
Planning the osteotomy line, device placement, and direction of the distraction

The planning can be done virtually (Fig. 2) using different available software [5, 8].

When performing preoperative planning, preliminary decisions regarding the osteotomy lines, the size of the bone flap, the direction of the distraction, and location of the distraction devices are made (Fig. 2). This helps to maximise intracranial volume gain while achieving as good aesthetic result as possible.

The osteotomy line in its upper part, crossing the skull on the coronal axis, must be placed as anteriorly as possible and occipitally it should be done as low as feasible. To gain maximal intracranial volume increase, it is preferred to place it below the venous torcula. When planning the osteotomy line, one needs to know what type of distractor devices will be used, since they vary in size and shape. It is crucial that the distractor devices can be fitted over the osteotomy line lying tight to the calvarial bone with the activation arms passing as close as possible to it. Additionally, the burr hole positions should be planned at this stage.

**Fig. 3:**
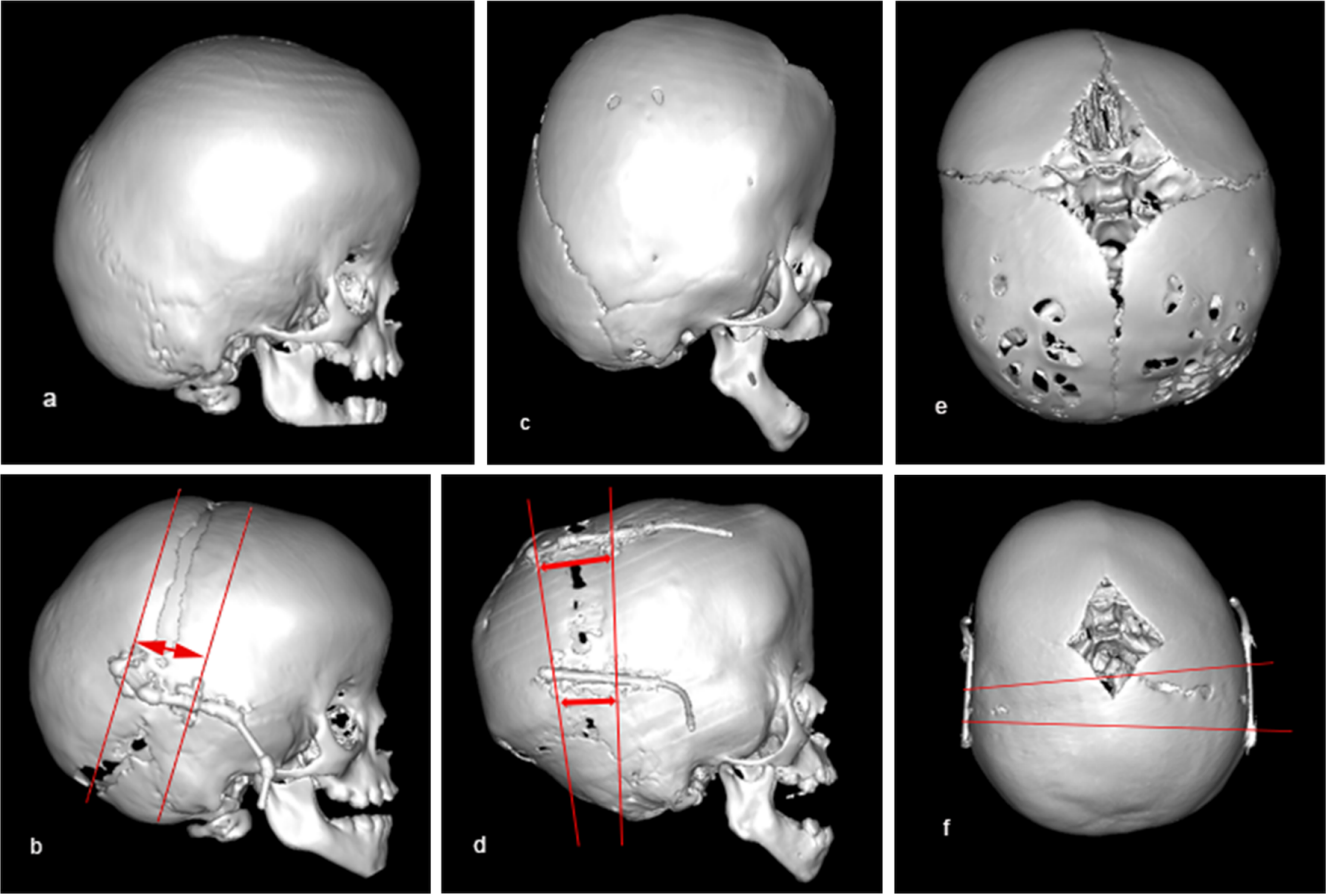
3D reconstructions of the skull CT images presenting different distraction options. Conventional PCVD with posterior shifting of the bone: **a** preoperative image and **b** image after the treatment was completed; PCVD with rotation of the bone flap along the lower occipital osteotomy line: **c** preoperative image and **d** image after the treatment was completed; asymmetrical PCVD in patient with bi-coronal and right lambdoid synostosis: **e** preoperative image and **f** image after the treatment was completed

Usually, the direction of the distraction is planned to go straight posteriorly, especially avoiding shifting of the bone flap downwards (Figs. 2 and 3b). This decision affects the intracranial volume increase and the shape of the skull achieved by the procedure.

It is possible to add a rotational component to the direction of distraction (Fig. 3b, c). In this technique, the osteotomised bone flap will be tilting during distraction, slightly rotating along its lower occipital osteotomy line. As a result, while the gap between the bony edges at the occipital area will remain small, it will be maximal up at the vertex (Fig. 3c). By doing so, a particularly good expansion is achieved with minimal cosmetic disadvantage in the occipital area [20].

Distraction could be done in an asymmetrical fashion if necessary (Fig. 3e, f) [6].

**Fig. 4:**
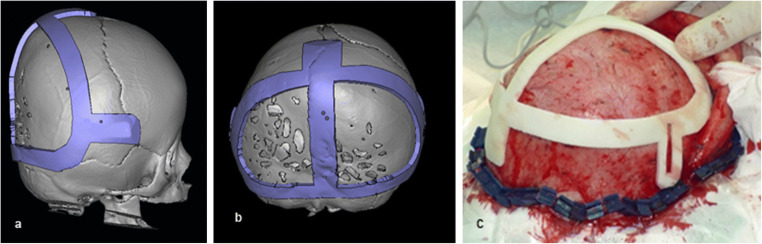
Individual sawing templates for intraoperative use: **a** and **b** virtual planning; **c** template being used during the operation

The authors recommend using individual rapid 3D-printed prototypes whenever possible for preoperative planning. On such a printed skull model, not only the position of the osteotomy lines could be planned, additionally, the distractors can be fitted and even moulded preoperatively. It is possible to 3D print individual sawing templates for intraoperative use (Fig. 4). This will ensure that the osteotomy will be performed exactly in accordance with the preoperative plan. Such guides can be manufactured “in house”, and they are commercially available. The role of prototypes and sawing guides may be highlighted when there is a shortage of experience with PCVD.

### Surgical armamentarium

Standard surgical equipment suitable for craniosynostosis surgery is required. They include bone rongeurs, dural dissectors, and motor drills with sufficient choice of attachments and tool heads (ball or rose head, a craniotomy attachment with tapered tool). When cutting a craniotomy line over venous sinuses, the authors use a piezoelectric drill, or bone rongeurs.

#### Distractor devices

The distractors are chosen according to the institutional preferences. There are several commercially available distractor devices, and some centres could have institutional “in house manufactured” distractors. The authors are not aware of any studies comparing different devices, so the choice is based on individual preference and availability. The devices vary in the shape of the footplate, heights of the profile, length of distraction provided, and construction of the bar connecting the footplates (allowing rotation between the footplates or not) and can have either a fixed or changeable distraction axis. Additionally, extension rods, used for activation of the devices, can be removable if the device is internal or fixed, making the devices internal-external.

For small infants, devices with lower profile should be chosen, even though such devices usually have lower distraction potential provided. The authors prefer internal devices with removable activation arms.

There should be self-tapered titanium screws, suitable for use with the distractor devices.

### Anaesthesiologic requirements

As the patients will be in the prone position, the anaesthesia and monitoring of the patients are challenging. The intubation tube must be safely secured. The authors prefer to use trans nasal intubation whenever feasible. Two venous lines are required, one for possible blood transfusion, another for anaesthetics and fluid infusion. The arterial cannula is placed for invasive blood pressure monitoring. Urine output should be monitored throughout the operation, and postoperatively. PCVD is a procedure that is associated with minimal intraoperative bleeding, when compared to other techniques in craniosynostosis surgery. Blood transfusions are rarely needed and the use of tranexamic acid is volitional. Despite this, there should be readiness for blood transfusion, whenever needed. Antibiotic prophylaxis is used according to the institutional guidelines for neurosurgical procedures with implant placement.

### Positioning

PCVD is performed with the patient in the prone position with the head slightly elevated above the level of the heart. A U-shaped headrest is used. If foramen magnum decompression is planned to be done, the patient should be positioned with the head being in a flexed position. The patients’ eyes and gentle facial skin should be protected when positioning the patient. The eyes are protected with pads. The authors protect prominent parts of the face from damage through friction during the operation by covering them with silicone tapes prior to the procedure. When preparing the operative area, alcohol solutions should be used carefully so as not to get them in the eyes or on the sensitive facial skin. The alcohol solution should not get onto the intubation tube fixation tapes due to a risk of accidental extubating of the patient. Subcutaneous injection of local anaesthetic with adrenalin prior to skin incision is used. When positioning the patient’s head on the headrest and draping, sufficient skin should be sterile draped and visible anteriorly to the planned incision allowing externalisation of the distractor’s activation arms at the end of the procedure. The direction of the distraction is drawn on the patient’s skin, prior to draping to ensure keeping to the preoperative plan when there will be less anatomical landmarks visible during the procedure.

## Surgical technique

The skin is opened in a bi-coronal weave fashion. The incision should be placed allowing surgery to the fronto-orbital area to be done later using the same incision, if necessary. The dissection of the skin is usually done leaving the periosteum attached to the bone to reduce the bleeding. The periosteum is incised only at the site of osteotomy lines. However, occipitally the periosteum is opened and dissection continued subperiosteally below the muscle insertion area to preserve muscle tissue. Alternatively, the muscles with the fascia should be cut several millimetres below their insertion area allowing suturing of them at the end of the procedure.

The osteotomy line is marked on the bone. This is done following the preoperative plan, using the abovementioned individual sawing templates, whenever available. In trained hands, expenses can be reduced by doing the drawing free hand.

The authors make the burr holes with a ball drill. At all stages, drilling should be performed extremely carefully, avoiding dural damage and keeping in mind that the inner surface of the bone is usually very uneven.

Burr holes are placed along the planned line bilaterally on the side of the sagittal suture, downwards temporally, and on both sides of the lambdoid suture. There must be placed as many burr holes as are necessary to provide careful dural dissection all the way along the future osteotomy line though keeping in mind that burr holes could interfere with device attachment.

**Fig. 5:**
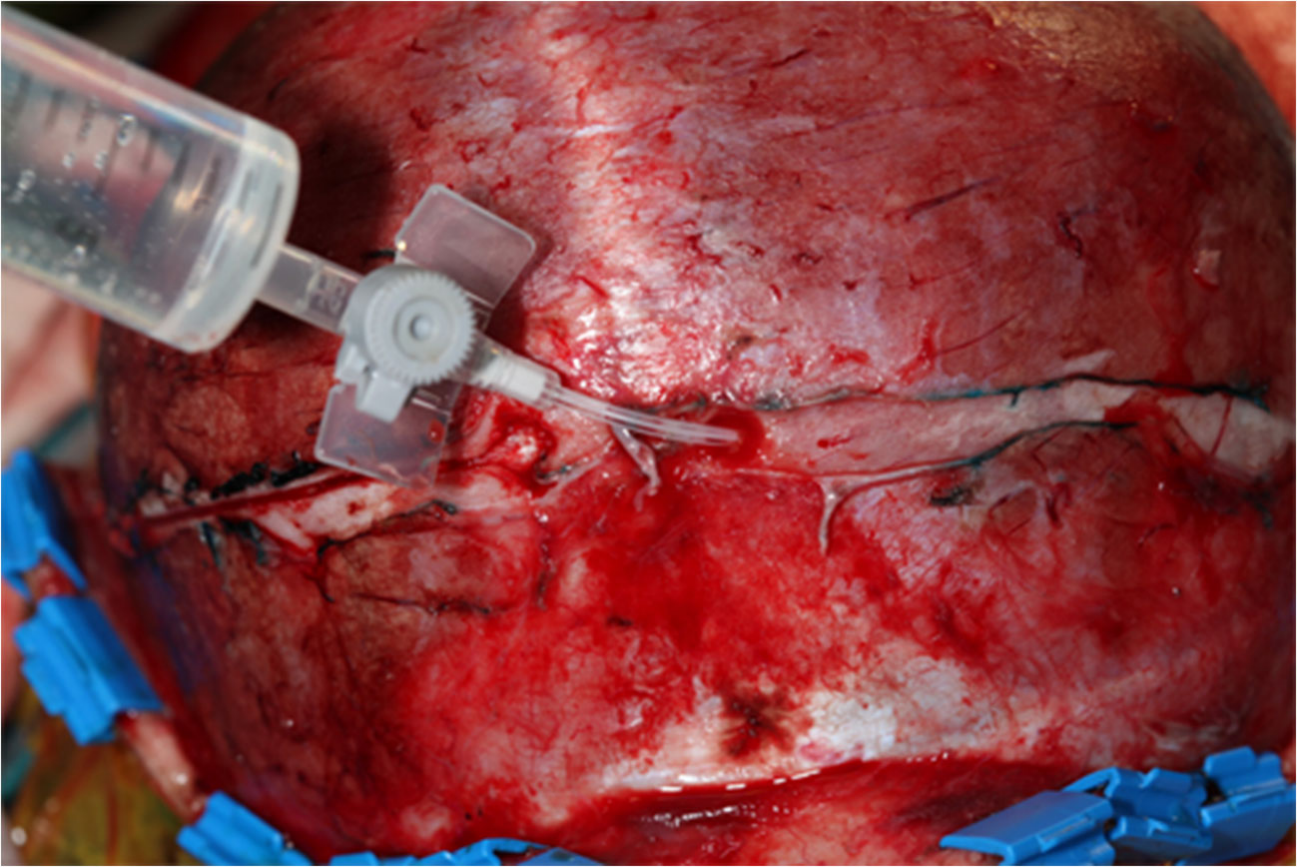
Confirming of the successful dural dissection between the burr holes using the saline “injection”

The dura is carefully dissected from one burr hole to another using dural dissectors (blunt and sharp ones). Saline “injection” between the bone and the dura helps to confirm successful dissection of the dura between the burr holes (Fig. 5).

**Fig. 6:**
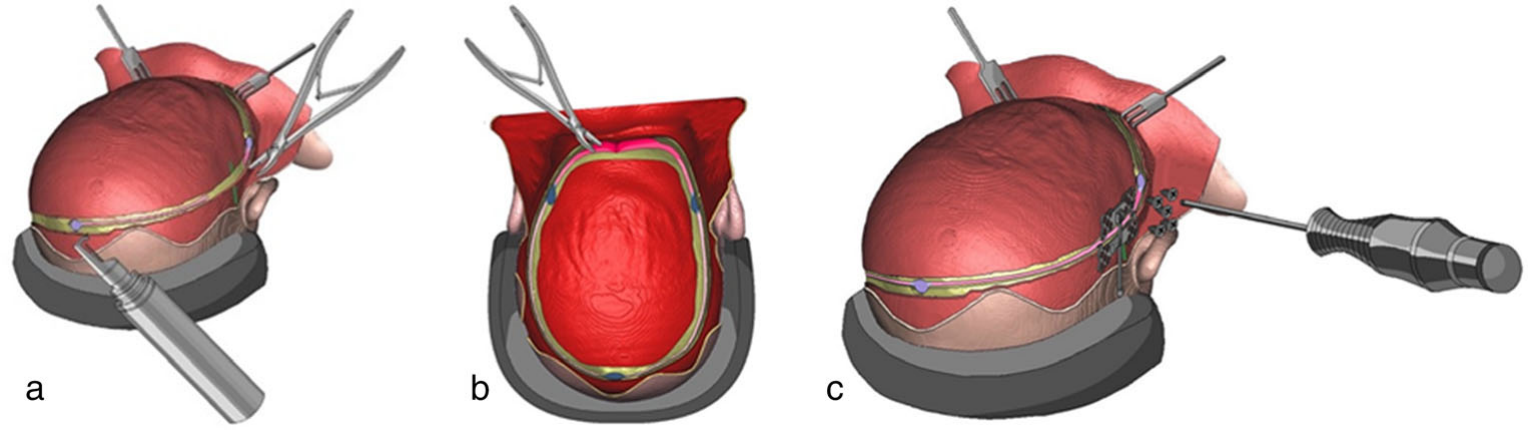
**a** and **b** Drawings presenting the cutting of the osteotomy. **c** Placement of the devices

Further craniotomy is performed in a highly controlled manner with a standard craniotome or with a piezoelectric drill. It is preferred to start doing craniotomy from the safest areas, usually temporally. Next the bone is cut over the sagittal suture and finally occipitally over the posterior sinus structures. The area of the lambdoid suture and the occipital osteotomy line below the venous torcula are the riskiest areas due to the proximity of venous sinuses and since the dura is usually strongly attached to the bone at the lambdoid suture. In the occipital area, bleeding is usual from emissary veins. This is dealt with using diathermy, bone wax, and the haemostatic matrix with thrombin. The osteotomy over the lambdoid suture and occipitally is performed using bone rongeurs; thus, a craniectomy, up to 5 mm wide, is created in this area (Fig. 6).

If the osteotomy line is not reaching low enough occipitally, barrel stave cuts to the occipital bone below the osteotomy line, with green stick fracturing of the bone could be done [6, 7]. If the patient has significant Chiari I malformation, it could be addressed at this stage by removing the posterior ring of foramen magnum with the usual occipital craniectomy. The dura should not be opened, to avoid any risk of CSF leakage.

To ensure stability of the bone flap during fixation of the distractors, a short bone bridge is left between the bones, usually temporally. This bridge is cut after the distractors are fixed.

### Selecting and fixation of the distractor

The number of distractors is chosen patient specific and by personal preferences. The authors use two to four devices [12]. The more devices used, the more stable the construct will be and better control of the distraction will be achieved. Nevertheless, that increases the cost and duration of the procedure and could be technically challenging.

**Fig. 7:**
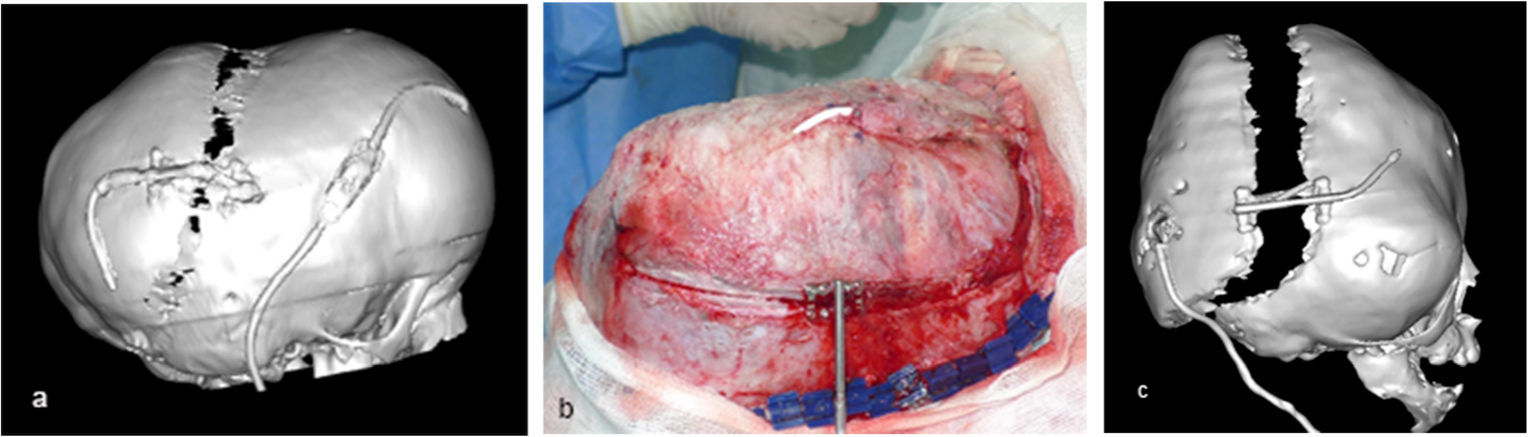
**A** Placement of the distraction devices when there is a ventriculoperitoneal shunt inserted to the anterior horn of the lateral ventricle. Notice that in some cases the shunt’s subcutaneous part should be relocated to avoid its extrusion during the distraction. **b** Intraoperative image of the ventriculoperitoneal shunt inserted into the posterior horn, the shunt is left partially unexposed during the procedure. Shown in **c** is a 3D CT image taken after the completion of the distraction when the shunt moves backwards with the bone flap

When placing devices, the following points should be kept in mind. The devices should be placed in accordance with the preoperative plan, so that the desired direction of distraction should be provided. The authors prefer to place the devices with the activation arms directed anteriorly (Fig. 6). An exception is made in cases where there is a shunt placed from the anterior horn (Fig. 7a).

All the devices should be placed in a parallel direction to avoid conflict of the vectors. The authors have developed novel guides for placement of the devices in as parallel direction as possible [11]. The devices should be placed as close to the bone as possible, to avoid tension to the skin. The footplates should be moulded, if necessary, to fit tight to the bone in the planned position. Taking into account the abovementioned aspects, increasing the number of devices to over two makes it more demanding to fit all devices tightly to the bone and in a parallel direction.

The authors prefer to use two devices placed temporally on both sides in smaller children and three devices, with the third one placed close to the midline rostrally on either side of the sagittal suture, if the procedure is performed on a child above a few years of age. When two distractors are placed, they should be positioned in the middle of the bone flap to provide pushing back of the whole flap and not hinging off it, except for cases where rotational direction of the distraction is chosen.

The distractors are fixed over the osteotomy line with footplates on both sides of it. At least four self-drilling, self-tapering titanium mini screws are used on each footplate. Additionally, biodegradable pins SonicWeld ® can be used to fix the devices replacing part or all of the titanium screws [2, 13]. When inserting the screws, the dura is protected from perforation by placing dissector between the bone and the dura.

After the distractors are fixed in place and the last bone bridge is cut, the activation arms are attached if an internal device is used. The activation arms are externalised, whenever possible, through separate small skin incisions. Next, the construct is tested by activating the devices for a few millimetres. During this testing, the free movement of the bone flap, especially occipitally, is confirmed, and direction of the distraction and parallelism of the devices are put to test. Testing of the devices prior to their implantation is recommended.

One could leave at this early stage a few millimetres of distraction, so that the posterior part of the bone flap has been moved over the occipital bone edge and some distraction is achieved. The devices could be placed so, that prior to their activation, there is a few millimetre gap at the osteotomy line, thus providing extra distraction distance additionally to that provided by the devices. Alternatively, the distractors should be closed tight after implantation with as close contact of the bone edges as possible to provide perfect conditions for the initialisation of the ossification [15].

Prior to skin closure, the soft tissue around the activation arms, especially the periosteum, is checked and trimmed if necessary, to avoid “grabbing” it around the rotating parts of the devices during the activation period.

**Fig. 8:**
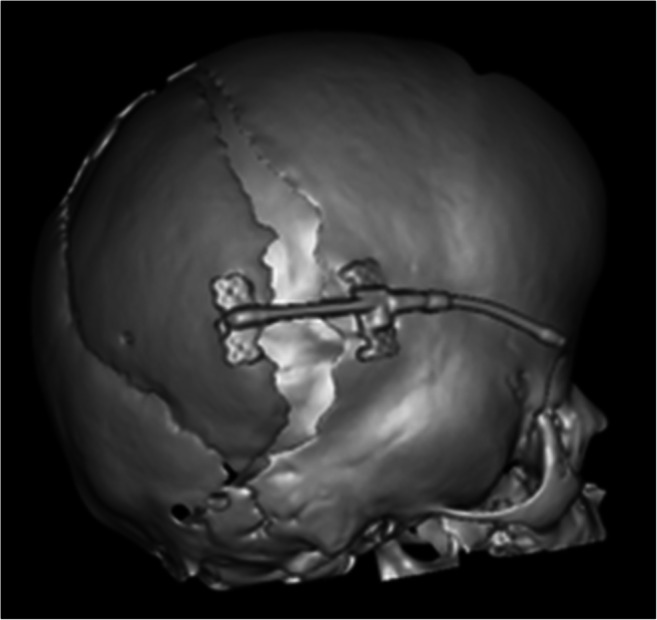
A 3D CT image showing the so-called gull wing complication, where the bone flap bends at the patent lambdoid sutures during the distraction

When performing the procedure in the abovementioned manner, the bone is left attached to the dura. In some patients, the bone flap should be detached from the dura and mobilised to be reinforced with biodegradable plates placed on the inner surface of the bone. This technique should be used when the bone is too thin for the screws alone to provide stable fixation of the devices [1]. Additionally, such reinforcement must be considered when there is a risk of bone flap bending and thus reducing the amount of intracranial volume increase achieved by the procedure. This being a risk in some patients not only due to the extremely thin bone, but, especially, if the lambdoid suture is opened widely (Fig. 8). This manoeuvre decreases the risk of dural damage by the screws in cases when the screws’ length exceeds the thickness of the bone. However, detaching of the bone flap increases the risk of bleeding from venous sinuses and inevitably increases operative time, especially in less experienced hands. However, according to the authors’ previous study, the full mobilisation of the bone flap does not hamper the ossification [12].

## Postoperative course

After the skin is closed, the location where the distractors’ arms penetrate the skin is covered with antiseptic (i.e. iodine) ointment. The bandages are placed under the activation arms and over them to protect the skin and the activation arms.

The patient is taken to either the postoperative recovery unit or to the intensive care unit, according to the institutional protocol for postoperative treatment after craniosynostosis surgery. The sedation is ceased, and the patient is extubated as soon as possible. As after every craniosynostosis surgery, the head position should be changed frequently to avoid pressure ulcers. Especially prolonged pressure to the skin over the devices should be avoided.

Blood transfusion is usually not necessary during and after these procedures. The blood tests and urine output are monitored postoperatively.

## Distraction protocol

**Fig. 9:**
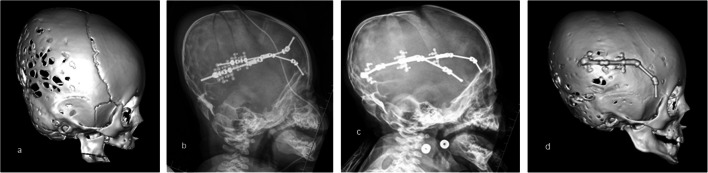
**A** Preoperative 3D CT image; **b** postoperative x-ray; **c** x-ray taken at the follow-up; **d** 3D CT image taken prior to removal of the devices, where ossification is visible

To ensure the position of the osteotomy, postoperative skull x-rays are taken prior to initialising of the distraction (Fig. 9a).

**Fig. 10:**
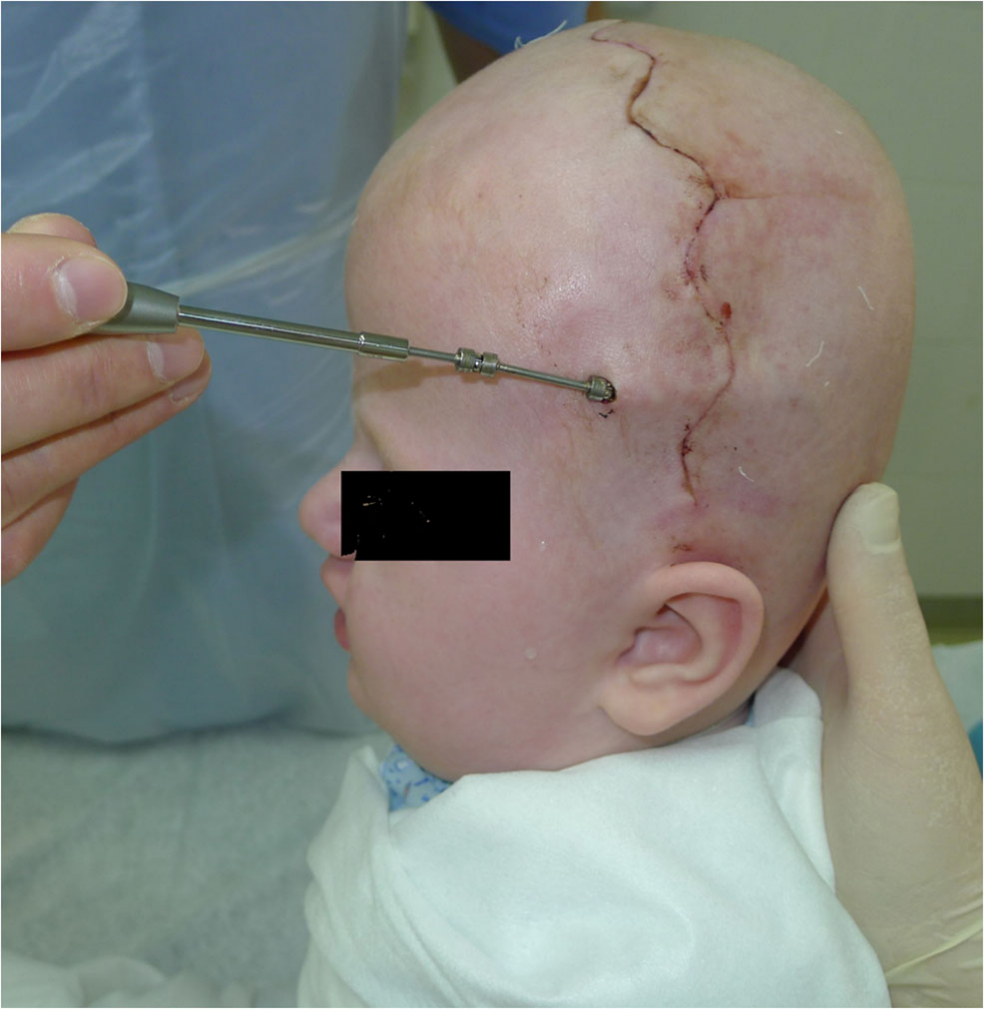
Performing the daily distraction

The latency period is 5 to 7 days, allowing the wound to heal before starting the distraction itself. The activation of the devices is done at a speed of 0.5–1 mm/day, in one or two sessions per day (Fig. 10). It can be performed at home by parents, after teaching them the routine. However, if the patient remains hospitalised and the procedure is performed by the surgeon, the speed can be faster due to daily monitoring of the skin condition [8]. During this period, the wound around the extruded activation arms should be cleaned and treated with antiseptic ointment daily. The whole process should be well documented. At the Burdenko Institute, Moscow, for example, special “Distraction protocol documentation” is used to be filled in by the parents and the medical staff.

The activation period lasts from 2 to 4 weeks, depending on the device used, planned distraction distance, and the chosen activation speed. During this time, the authors recommend weekly follow-up visits for monitoring of the skin and device conditions. The authors prefer to perform lateral x-rays at such visits to monitor progression of the activation (Fig. 9b, c). Additionally, ultrasound could be used for such monitoring [15]. Activation becomes more difficult with time due to resistance. If serious problems occur, parents should contact the unit for assistance.

The detachable activation arms are removed after the desired distraction distance is achieved. This may require anaesthesia depending on the type of the attachment system of the devices. When external-internal devices are used, the external part could be cut at this stage to internalise the devices [4]. The last few millimetres of activation can be completed during the same anaesthesia prior to removing of the activation arms, if necessary.

The distractor devices are left under the skin for the ossification period of at least 1 month in very small children and up to 6 months in older children. Prior to removal of the devices, the sufficient ossification of the bony gap should be confirmed by plain x-ray, skull CT, or ultrasound (Fig. 9d) [9, 15]. The ossification period should not be prolonged unnecessarily since the devices can become covered by the bone.

**Fig. 11:**
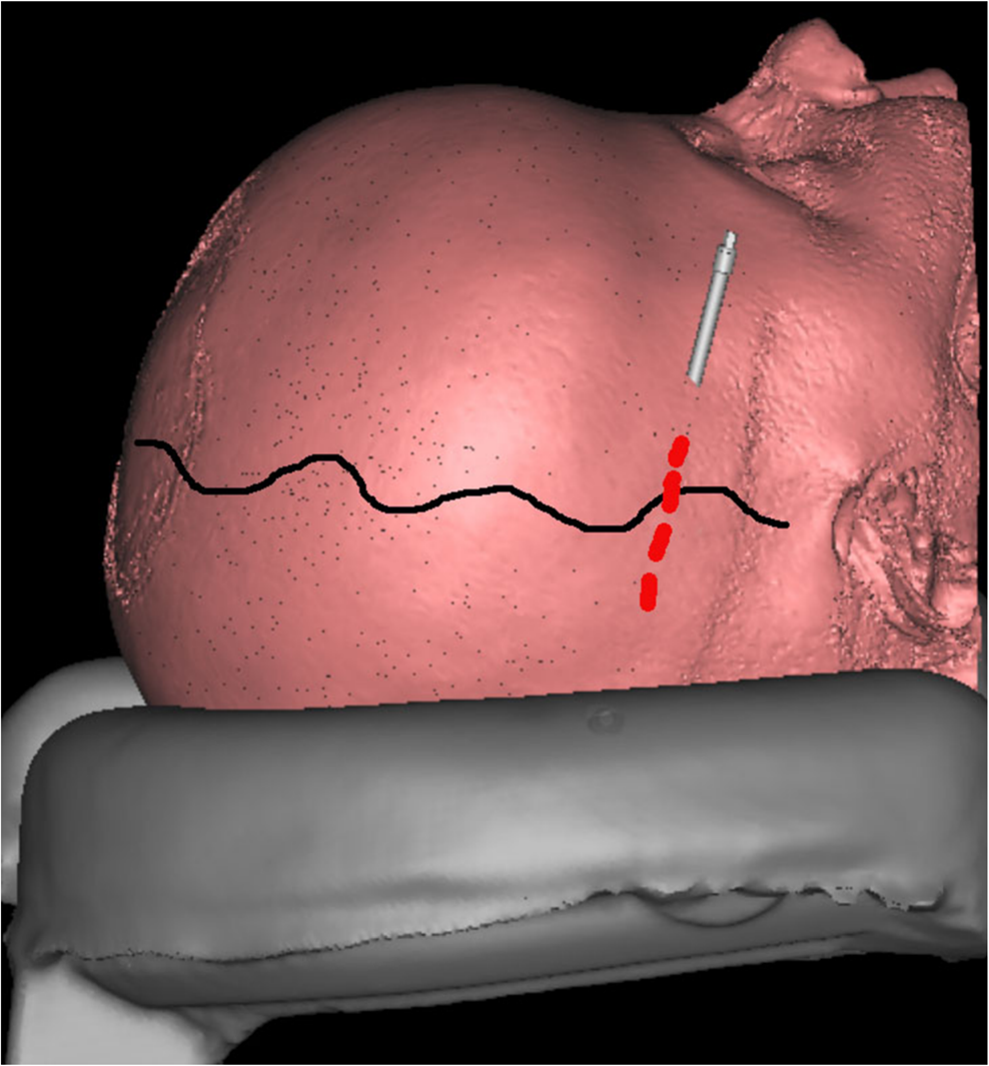
Drawing proposing the location of the skin incision used for the device removal procedure. The separate incision is placed over the device itself usually perpendicular to the bi-coronal incision used during the PCVD operation itself

The removal of distractors could be easily performed from separate small skin cuts done over the devices usually perpendicular to the bi-coronal incision done during the PCVD operation itself (Fig. 11). When using resorbable pins to fixate the devices, removal is technically easier [13].

## Tips and tricks how to avoid complications

The PCVD procedure requires commitment and patience of both the surgeon and the family, during several weeks after the surgery itself. These procedures are associated with many minor problems that can require either conservative or surgical treatment. However, serious complications are rare [4, 12, 18, 19].

**Fig. 12:**
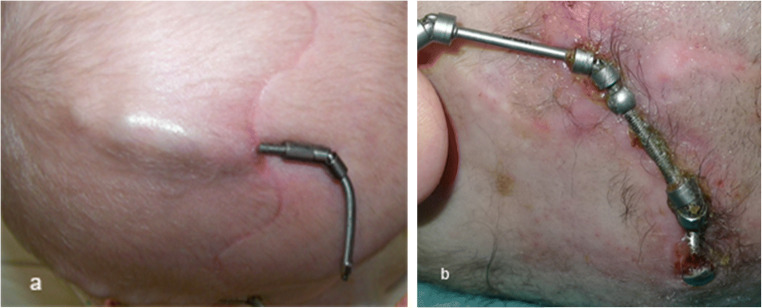
Photos showing **a** healthy skin over the device. **b** Complication with extrusion of the device through the skin

Minor skin problems are usual (Fig. 12). Excessive tension of the skin can be avoided using devices with as low profile as possible and by thoroughly positioning the devices (see “Surgical technique”).

The exit area of the external arms must be carefully cleaned and covered with local antiseptics always prior to the device activation. Before discharging the patient, parents should be instructed thoroughly on how to do distraction and how to take care of the wound.

If the activation process is started too early or carried out too fast, there will be a risk of wound dehiscence. The activation could be paused for few days or its speed decreased, if necessary.

In cases when, despite of the abovementioned measures, the distractor device penetrates the skin (Fig. 12b), local revision and local antiseptic care can usually be sufficient to manage the problem until the planned removal of the distractor devices is performed. However, sometimes the device should be re-installed to a better position for the activation to be continued. When the desired amount of distraction is achieved, the devices can be replaced by resorbable plates, if maintaining them until the end of ossification is impossible due to skin problems.

The distraction devices are under significant stress during the whole process and thus there is a potential risk of their loosening or breaking. This could be minimised by increasing the number of devices and choosing ones with a higher profile [12, 14, 19]. However, as mentioned earlier, this increases the risk of skin problems. Thus, one should find suitable devices for each patient individually, taking into account the patient’s age, skin condition, skull shape, and bone thickness.

The external activation arms could fracture, especially if they are flexible. If detachable ones are used, they can easily be replaced; otherwise, a whole new device should be re-inserted.

If there is dural damage during the operation, the dura should be sutured in a watertight manner since a dural tear, even if patched carefully, might later result in CSF leakage during the postoperative course. If a dural tear has occurred, the authors prefer a delayed latency time before distraction is started; otherwise, there is a risk of re-opening of the tear. There is some risk of postoperative dural and/or vascular tears during the activation period. The screws can extend beyond the bone and damage the dura during the activation period.

Postoperative CSF leakage must be always actively addressed. Temporary lumbar drainage placement is often curative though revision surgery for repair of the dural tearing can be required. Remember, “prevention is better than cure”.

## Summary of important steps

To ensure successful and safe PCVD, the authors stress the following points:

1. Before PCVD surgery, careful preoperative planning is necessary
  - Plan the osteotomy line and burr holes
  - Plan the distraction direction
  - Choose the distraction devices and plan their positioning
  - Is an individual rapid 3D-printed prototype or sawing guide(s) required? Order them in advance.
2. Carefully position and drape the patient. Remember to leave space for the extrusion of the distractor external arms from separate incisions.
3. Be extremely careful when drilling and cutting the bone. Dural tears are hazardous in PCVD. Prevention is better than cure!
4. Check that the bone flap can move over the occipital bone edge freely.
5. Place the devices in a parallel direction. Do not use more devices than necessary. Usually, two devices are sufficient.
6. Check that the periosteum is not grabbed by the activation arms.
7. Check that there is no skin tension at the exit areas of the activation arms.
